# Protein Data Bank Japan: A unified portal for integrating structural and chemical data to explore protein–ligand interactions in PDB and PubChem

**DOI:** 10.1002/pro.70702

**Published:** 2026-07-08

**Authors:** Gert‐Jan Bekker, Chioko Nagao, Satomi Niwa, Genji Kurisu

**Affiliations:** ^1^ Institute for Protein Research University of Osaka Osaka Japan; ^2^ JBiC Research Institute Japan Biological Informatics Consortium (JBiC) Tokyo Japan; ^3^ Protein Research Foundation Osaka Japan

**Keywords:** chemical compounds, data archives, PDB, protein structure, PubChem

## Abstract

Protein Data Bank Japan (https://pdbj.org/) is the Asian hub of three‐dimensional (3D) macromolecular structure data and a founding member of the global Protein Data Bank (PDB) network. Over two decades, we have curated and distributed experimentally determined structures, complementing international collaborations with Research Collaboratory for Structural Bioinformatics (RCSB) PDB, Biological Magnetic Resonance Data Bank, Protein Data Bank in Europe (PDBe), and Electron Microscopy Data Bank. In response to user demand for integrated structural and chemical data, we developed a new PubChem Portal that enables interactive exploration of compound‐protein interactions. Users can view ligand binding poses in 3D via our Web Graphics Library (WebGL)‐based Molmil viewer, with key interactions highlighted and key residues displayed in semi‐transparent stick models, enhanced through integration with secondary databases (e.g., Dynamics DB, eF‐site) for advanced insights into molecular dynamics and electrostatics. The system supports filtering by UniProt ID, Enzyme Commission (EC) number, Pfam ID, or PROSITE ID to identify structurally related compounds and visualizes protein–ligand interactions. A dynamic two‐dimensional (2D) Japan Agency for Medical Research and Development representation enables real‐time atom‐level navigation, with clickable atoms linking to 3D structures. This tool allows users to explore compound‐protein interaction landscapes, identify potential binding modes, and guide experimental design, such as mutagenesis or crystallization. The portal offers a comprehensive, user‐centered ecosystem that bridges chemical and structural data, enhancing access to biological insights through integrated visualization and analysis.

## 1 INTRODUCTION

The rapid expansion of biological data has transformed structural biology from a discipline grounded in isolated experimental observations into a field increasingly reliant on integrated, multi‐dimensional data ecosystems. Central to this transformation is the need for seamless interoperability between diverse databases, including protein sequences, three‐dimensional (3D) structures, genomic variation, and chemical compounds, to enable holistic understanding of molecular function, interactions, and dynamics.

The 3D structural data of biological macromolecules are collaboratively maintained by the worldwide Protein Data Bank (wwPDB) partnership (Burley et al., 2019). Protein Data Bank Japan (PDBj, https://pdbj.org) has accepted and processed the 3D structure data of biological macromolecules from Asia and Oceania and distributed the globally collected data since 2000 (Kurisu et al., 2022). In total, roughly 21.8% of all Protein Data Bank (PDB) entries have been processed by PDBj as of the end of 2025. Since its founding, PDBj has developed a suite of original services, including the recently introduced UniProt Portal (Bekker et al., 2025a, 2025b), which integrates protein sequences with PDB structures and genomic variation data from japanese multi‐omics reference panel (jMorp) and medical genomics japan variant database (MGeND), establishing a foundation for integrated structural data access.

While the PDB remains the cornerstone of experimental structural biology, its utility is significantly enhanced when contextualized with other critical biological data sources. For instance, small molecule ligands bound to proteins are not static annotations in PDB entries; rather, they represent dynamic interaction landscapes that inform drug design, enzyme mechanism elucidation, and disease pathogenesis. However, current access to such data is often fragmented: ligand information is scattered across PDB entries, with limited contextualization of chemical properties, biological activity, or interaction partners. Similarly, users frequently face challenges in identifying the most relevant structure for a given protein due to the presence of multiple overlapping entries with varying resolutions, partner molecules, and experimental conditions.

To address these limitations, we have developed a new integrative service, the PDBj PubChem Portal, designed to bridge the gap between chemical compound data from PubChem and structural data from the PDB. This portal enables researchers to explore protein–ligand interactions in a unified, interactive environment, offering both structural visualization and rich contextual metadata. By integrating PubChem's extensive chemical database, which includes over 150 million chemical substances with detailed physicochemical, biological, and safety profiles, with high‐resolution PDB structures, the PubChem Portal offers a powerful platform for identifying, analyzing, and interpreting molecular interactions.

The development of this service was informed by direct feedback from the scientific community, collected via questionnaires during luncheon seminars at major national biological conferences (Bekker et al., 2025a). Users consistently emphasized the need for tools that facilitate cross‐database integration, particularly between chemical data and structural information. Bioinformaticians and structural biologists expressed a strong desire to combine genomic, pathway, and chemical data to build predictive models of molecular behavior. Moreover, they highlighted difficulties in selecting appropriate PDB entries for a given compound or protein, often due to a lack of clear criteria for structure selection or insufficient visualization of protein–ligand interactions.

Building upon our earlier efforts in structural data integration, notably the UniProt Portal (Bekker et al., 2025a, 2025b), which linked protein sequence data with PDB structures and genomic variation data from jMorp and MGeND, we now extend this integrative philosophy to chemical compounds. The PubChem Portal introduces a novel framework for interactive visualization of ligand binding sites, intermolecular contacts, and interaction patterns across multiple PDB entries. It further enables users to filter and analyze data based on biological classifications (UniProt ID, EC number, Pfam ID, or PROSITE ID), providing insights into the functional and evolutionary context of ligand binding.

This paper first presents a comprehensive overview of the PDBj ecosystem, detailing our curated archives, secondary databases for derived insights, and essential tools, with a detailed overview of our archives, tools and services in Table 1. Building upon this foundation of robust data integration, previously exemplified by the UniProt Portal, we now describe the design, implementation, and evaluation of our latest addition: the PubChem Portal. As a new service within the ecosystem, it bridges chemical compound data from PubChem with structural data from the PDB to enable interactive exploration. We detail its core functionalities, including 3D/2D visualization, contact mapping, biological classification, and batch retrieval, and demonstrate how this tool facilitates a transition from static structure views to dynamic, context‐aware molecular understanding for guiding mutagenesis studies and crystallization strategies.

**TABLE 1:** Overview of Protein Data Bank Japan (PDBj)‐developed archives, services, and tools.

Service name	URL
*Search PDB (PDBj Mine)*	pdbj.org/search/pdb‐filter
A centralized relational database interface allowing users to search all PDB entries by ID or keywords. It consolidates metadata from core archives (PDB, EMDB), secondary databases (EMPIAR), and validation reports into a single queryable system via graphical or structured query language (SQL) interfaces.	
*Chemie search*	pdbj.org/chemie‐search
A tool for searching the Chemical Component Dictionary (chem_comp) within the PDB ecosystem. Users can filter compound libraries, view structures in 3D viewers like Molmil, and link compounds to relevant protein entries.	
*Search BMRB*	bmrbj.pdbj.org
A service dedicated to querying the Biological Magnetic Resonance Data Bank (BMRB) for biomolecular NMR structural data and spectra.	
*Sequence‐Navigator*	pdbj.org/seq‐navi
A tool designed for users who start with only an amino acid sequence to find structures in the PDB with similar sequences. It serves as an entry point for exploring structural biology data without requiring prior knowledge of specific 3D structures beyond the protein sequence itself.	
*EM Navigator/Yorodumi*	pdbj.org/emnavi
An integrated website providing a user‐friendly interface to view, rotate, analyze, and learn about biological molecules from PDB, EMDB, and chemical component databases. It features unified visualization capabilities with specialized tools for 3D electron microscopy Cryogenic Electron Microscopy (cryo‐EM) data analysis.	
*Omokage search*	pdbj.org/omokage
A shape similarity search service that identifies structurally similar macromolecules by comparing overall molecular shapes while ignoring atomic details, applicable to both PDB and EMDB entries.	
*wwPDB/RDF*	rdf.wwpdb.org
A collection of wwPDB data formatted in Resource Description Framework (RDF), enabling semantic web integration with an accompanying Web Ontology Language ontology for standardized gene/genome/sequence/disease/drug relationships.	
*jV: Graphic Viewer*	pdbj.org/jv/
An interactive 3D viewer program (historically a Java applet, now often referenced alongside modern viewers) designed to visualize the structures of proteins and nucleic acids deposited in PDB or EMDB.	
*Molmil: WebGL Molecular Viewer*	pdbj.org/molmil2/
A high‐performance, plugin‐free web‐based molecular visualization tool supporting various formats (PDB, mmCIF, mmJSON). It enables interactive 3D rotation/rendering and links to secondary data like electron density maps and dynamics simulations.	
*NMRToolBox*	bmrbj.pdbj.org/en/nmr_tool_box.html
A suite of tools specifically designed for spectral assignment and analysis of NMR data associated with BMRB entries.	
*gmfit*	pdbj.org/gmfit/
A service utilizing Gaussian mixture models to perform fast 3D fitting between two objects, such as aligning PDB structures onto EM density maps or comparing structural ensembles.	
*HOmology Modeling of Complex Structure*	homcos.pdbj.org
A service for searching 3D complex structures in the PDB based on input sequences or chemical ligands, followed by modeling these complexes using identified template structures to predict binding sites.	
*eF‐site*	pdbj.org/eF‐site/
A database displaying electrostatic potentials and hydrophobic properties on the Connolly surfaces of protein functional sites, aiding in the analysis of molecular recognition mechanisms.	
*eF‐seek*	pdbj.org/eF‐seek/
A server that searches for ligand binding sites similar to a user‐uploaded PDB coordinate file using an algorithm based on clique search within the eF‐site database representatives.	
*eF‐surf*	pdbj.org/eF‐surf/
A web server calculating and visualizing the molecular surface of uploaded PDB files, generating electrostatic and hydrophobic maps consistent with those in the eF‐site database.	
*ProMode Elastic*	pdbj.org/promode‐elastic
A database providing Normal Mode Analysis (NMA) results for PDB entries using an elastic network model to describe flexibility and motion at the residue level.	
*Molecule of the Month*	numon.pdbj.org/mom/
An educational series introducing biological macromolecules, originally authored by Dr. David S. Goodsell of RCSB PDB, aimed at general audiences interested in structural biology, with Japanese, Chinese and Korean translations offered by PDBj.	
*Games*	numon.pdbj.org/games/
Educational tools (e.g., Pelmanism, Snake) designed to teach users about amino acids and proteins through interactive gameplay suitable for students or beginners.	
*Papermodels*	numon.pdbj.org/papermodel/
A service providing downloadable 3D paper models of biological macromolecules based on their deposited structures.	
*OneDep (Deposition to PDB, EMDB, or BMRB)*	deposit‐pdbj.wwpdb.org/deposition
The unified global deposition system used by all wwPDB partners (RCSB, PDBe, PDBj) to submit data for the PDB, EMDB, and BMRB archives simultaneously from a single interface.	
*Format Conversion*	mmcif.pdbj.org/converter/
Tools facilitating bidirectional conversion between legacy text‐based PDB formats and modern mmCIF/PDBx/mmJSON standards to ensure interoperability.	
*PDBx/mmCIF editor*	pdbj.org/cif‐editor/
A web‐based tool for locally editing and validating structural files in the PDBx/mmCIF format before deposition or analysis.	
*EMPIAR‐PDBj*	empiar.pdbj.org
An archive maintained by PDBj acting as a mirror of the Electron Microscopy Public Image Archive (EMPIAR), focusing on entries related to PDB structures with a customized interface for Asian/Oceanian users and HDD submission support.	
*BSM‐Arc*	bsma.pdbj.org
A unique archive by PDBj dedicated to preserving computational structural data, including molecular dynamics trajectories and models, distinct from raw experimental measurements.	
*XRDa*	xrda.pdbj.org
An archive specifically for preserving raw diffraction images (from x‐ray, electron, or neutron sources) used in crystallography, filling a critical gap by storing the primary imaging data rather than just final refined structures.	
*UniProt Portal*	pdbj.org/uniprot/
A service integrating protein sequence data from UniProtKB with structural information from PDB and AlphaFold models, allowing users to view sequences linked directly to 3D structures, ligand sites, variants, and genomic variations.	
*Sequence Navigator Pro*	pdbj.org/seqnavipro
An advanced extension of Sequence‐Navigator that integrates searches across multiple databases (PDB, Swiss‐Prot, AFDB) starting solely from an amino acid sequence, offering detailed predictions on disorder, secondary structure, hydropathy, and crystallization conditions.	
*Dynamics DB*	bsma.pdbj.org/dynamicsdb/
A database analyzing the stability of PDB entries using molecular dynamics simulations to provide per‐residue stability scores (*R*‐values), contact numbers, and insights into protein–ligand complex stability visualized in 3D structures.	
*PubChem Portal*	pdbj.org/pubchem/
An integrative service that centers on chemical compounds (PubChem) rather than individual PDB structures to explore protein–ligand interactions. It enables interactive 3D/2D visualization via Molmil, filters results by biological classifications (UniProt ID, EC number, Pfam, PROSITE), and aggregates cross‐entry interaction patterns to identify recurring binding motifs for guiding experimental design.	

Abbreviations: AFDB, AlphaFold Database; BSM‐Arc, Biological Structural Model Archive; EMDB, Electron Microscopy Data Bank; PDB, Protein Data Bank; wwPDB, worldwide Protein Data Bank; XRDa, Xtal Raw Data Archive.

### 1.1 Archives maintained by PDBj

PDBj serves as a central hub in the global wwPDB ecosystem, co‐maintaining three core archives, PDB, Electron Microscopy Data Bank (EMDB), and Biological Magnetic Resonance Data Bank (BMRB) (Hoch et al., 2023), with partners RCSB PDB (USA) and PDBe (Europe). For EMDB, it collaborates with European Molecular Biology Laboratory ‐ European Bioinformatics Institute (EMBL‐EBI) and RCSB PDB; for BMRB, it jointly manages the archive via its BMRBj initiative. All depositions pass through the shared OneDep system, enabling seamless global submission. Representing Asia and recently Oceania, PDBj processes approximately 21.8% of worldwide depositions. Beyond core efforts, PDBj maintains specialized archives supporting structural data lifecycles. Since 2018, it has hosted a mirror of the Electron Microscopy Public Image Archive (EMPIAR) (Bekker et al., 2022) (EMPIAR‐PDBj), focusing on PDB‐related entries with a customized interface and accepting hard disk drive (HDD) submissions from Asia for EMBL‐EBI integration.

To strengthen preservation, PDBj established two unique archives: the Biological Structural Model Archive (BSM‐Arc) (Bekker et al., 2020) and the Xtal Raw Data Archive (XRDa) (Bekker et al., 2022, 2025b). Together, they capture raw data across all structural methodologies and computational models. Depositors can register via ORCiD, collaborate on shared access, edit metadata through a web interface, and upload files efficiently using parallel transfers or rsync. Each entry receives a persistent identifier immediately upon creation, ensuring its Digital object identifier is fixed at deposition and becomes active upon release. Launched in 2018, BSM‐Arc hosts 72 entries totaling 61.8 TB of computational data (as of May 2026), including molecular dynamics trajectories, with structured files viewable directly in the integrated Molmil viewer. XRDa preserves raw diffraction images from macromolecular and chemical crystallography (x‐ray, electron, neutron), filling an Asian gap with 291 entries and 12.9 TB of data (as of May 2026). XRDa is lightly integrated with OneDep, where new PDB entries automatically appear in XRDa, while older records can be manually linked upon request. PDB‐linked XRDa entries are co‐published with corresponding PDB entries (Hold for publication status) or made immediately available.

PDBj further offers secondary databases providing derived insights: ProMode Elastic delivers weekly Normal Mode Analysis updates on protein dynamics (Wako et al., 2004) and eF‐site maps electrostatic potentials to functional sites (Kinoshita & Nakamura, 2004). Dynamics DB analyzes stability for over 23,962 active PDB entries (as of May 2026) using molecular simulations (Bekker et al., 2025b), generating per‐residue *R*‐values and contact number data. We have recently expanded this resource to include 7524 protein–ligand complexes executed via high‐performance computing infrastructure (HPCI) computational resources. This extension enables the analysis of interaction stability between ligands and their receptors, providing insights into binding dynamics and potential off‐target interactions directly within the viewer. These secondary databases are also integrated into our UniProt Portal and our new PubChem Portal.

As the only wwPDB partner systematically collecting raw experimental data across all modalities and computational sources, specifically diffraction images via XRDa and models via BSM‐Arc, PDBj ensures long‐term preservation, enhances reproducibility, and supports foundational research and advanced analysis by enabling validation beyond final deposited models. To facilitate access to these resources, we have developed a suite of tools for searching, visualization, and analysis.

### 1.2 Tools and services developed by PDBj

We maintain a comprehensive suite of tools and services to support exploration, analysis, and understanding of structural biology data, extending beyond our primary and secondary archives. These services are designed to empower users, from researchers to novices, with intuitive interfaces, powerful query capabilities, and deep integration across multiple data sources.

At the core of our offering is PDBj Mine, a centralized relational database (RDB) containing metadata for all PDB entries, including chemical components via the peptide‐like reference dictionary / biologically interesting molecule reference dictionary (PRD/BIRD) dictionary. This RDB also includes EMDB and EMPIAR data in both mmCIF and mmJSON formats, alongside file statistics, obsolete entries, and intermolecular contact pairs. By consolidating this data, Mine enables highly precise queries via a user‐friendly graphical interface or direct SQL access (via Representational State Transfer Application Programming Interfaces). Complementing this structural metadata is our Chemie service, dedicated to chemical compound exploration; users can search the PRD/BIRD dictionary, view structures in Molmil, and link compounds directly to PDB entries through quick search or advanced query tools. For individual PDB structure exploration, the Mine web interface provides detailed entry descriptions. Our molecular viewer, Molmil (Bekker et al., 2016), supports visualization of asymmetric units, biological assemblies, and electron density maps. Recently introduced is a 2D interactive topology view that simplifies complex structural relationships (Bekker et al., 2025a). For cryo‐EM entries, quality assessments are linked to the DAQ‐Score Database (Nakamura et al., 2023; Terashi et al., 2022). While wwPDB recently introduced Q‐score for atomic fit, DAQ‐score leverages deep learning to detect sequence‐to‐structure misalignments and conformational errors, offering complementary evaluation of model quality in low‐resolution structures by analyzing local density and fit. We have also enhanced the presentation of experimental details (e.g., crystallization conditions, refinement procedures), particularly for crystallographic structures and structures derived via integrated or hybrid methods (PDB‐IHM).

Molmil, a WebGL‐based viewer developed since 2013, serves as both a core component of our services and a standalone tool (Bekker et al., 2016). It supports loading of user‐provided structures and molecular dynamics trajectories without installation. An installable version, molmil‐app, is available for shell‐based or headless processing, which is also used for generating our PDB entry images (Bekker et al., 2022) and preparing our MD simulations for the Dynamics DB. In parallel with the viewer's development, we introduced PDBx/mmJSON, a compact, javaScript object notation (JSON)‐based format derived from the mmCIF dictionary. It maintains full semantic consistency with mmCIF, is more accessible to programming environments, and reduces the file size by approximately 36% (as of May 2026). A REST service allows users to retrieve mmJSON data for selected categories, facilitating easy integration into scripts and applications.

Most recently, we launched a new UniProt‐based portal unifying protein sequences from UniProtKB with structural data from PDB and AlphaFold (Bekker et al., 2025a). For each entry, it displays linked 3D structures highlighting secondary structure, ligand/glycosylation sites, and genomic variants. Clicking residues highlights them in 3D view with real‐time access to secondary archives (ProMode‐Elastic, Dynamics DB, eF‐site), enhanced with DAQ‐Score coloring, or compared with AlphaFold Database (AFDB) models. Supplementary panels provide summaries of PDB coverage, resolution, sequence match, mutation data, and validation to support structure selection and interpretation. Additionally, the Sequence Navigator Pro service explores databases starting from a protein amino acid sequence only, integrating searches across PDB, Swiss‐Prot, and AFDB (Bekker et al., 2025b). It delivers detailed results including sequence coverage, experimental details, purification methods, crystallization conditions, and primary literature links, along with predictions for secondary structure, disordered regions, and hydropathy. Users can also perform PubMed‐based keyword searches to contextualize findings.

Together, our services form a cohesive, accessible, and increasingly intelligent ecosystem for exploring and analyzing structural biology data, bridging sequence, structure, and genomic information to accelerate discovery and interpretation. Building upon this robust foundation, we now describe our latest development: the PubChem Portal.

### 1.3 Integration of structural compound data and chemical databases

In prior work (Bekker et al., 2025a, 2025b), we gathered user feedback via luncheon seminars highlighting the need for cross‐database integration. Users consistently prioritized linking structural data with external sequence databases and expressed strong interest in combining protein structures with genomic, variant, pathway, or chemical compound data. Additionally, users struggled to select appropriate structures among multiple entries for the same protein. To address these challenges, we developed the UniProt Portal service. Another requested feature was integration of chemical data from the PDB with chemical databases. While all wwPDB partners share foundational tools such as the Chemical Component Dictionary (CCD), BIRD, and OneDep validation pipelines, RCSB PDB focuses on coupling chemical data with macromolecular metadata via advanced graphical interfaces for structural constraints (Burley et al., 2021), whereas PDBe emphasizes biological context through automated annotation pipelines (Choudhary et al., 2025). In contrast, PDBj distinguishes itself by delivering unmatched data‐mining flexibility, allowing power users to execute custom SQL queries across all archived attributes simultaneously via our Mine RDB, while also providing accessible graphical links from search results for broader usability through the Chemie user interface. Building on this robust integration framework, the need to understand ligand behavior and chemical function drove the development of a new portal. The PDBj PubChem Portal enables direct exploration of compound‐protein interactions using a compound‐as‐organizing‐principle, where the portal is centered on PubChem entries rather than individual PDB structures. This approach facilitates unique capabilities, including cross‐entry interaction aggregation to identify recurring binding motifs across multiple structures, and secondary‐database overlays from Dynamics DB, eF‐site, and EDMap that provide real‐time insights into stability, electrostatics, and electron density directly within the viewer. It is important to note that while the tools provide rich, interactive access to structural and chemical data, their utility depends on the quality and completeness of the underlying PDB and PubChem data. For example, ligand annotations may be incomplete or inconsistent, and some interaction patterns may lack experimental validation. These limitations highlight the need for continued curation and cross‐referencing with experimental sources.

To implement this compound‐centered framework, we leveraged PubChem as the primary chemical reference database for linking PDB chemical components to external chemical information. Given that PubChem is one of the largest public chemical databases, covering small molecules, lipids, and nucleotides, we used it as a foundation for linking compound data with PDB structural information. PubChem is an open, publicly accessible chemical database hosted by the National Institutes of Health (NIH) (Kim et al., 2025). It serves as a comprehensive resource for chemical substances and their biological activities, mostly focusing on small molecules but also including larger ones like lipids and nucleotides. The database is organized into several collections, including PubChem Substance, PubChem Compound, and PubChem BioAssay, which contain information submitted by hundreds of data sources worldwide. Users can search by name, structure, or other identifiers to access comprehensive data on chemical properties, safety, and toxicity. All PubChem entries are stored in a PostgreSQL database as both molecular fingerprints (for efficient structural similarity searches) and RDKit objects (for computational processing). Similarly, chem_comp entries, representing chemical components in PDB structures, are also stored in the same database in both formats. While PubChem does not provide atomic coordinates, PDB chem_comp records contain them and are matched using RDKit's normalized molecular representations, derived from both PubChem's isomeric simplified molecular‐input line‐entry system (SMILES) and the 3D structural data in chem_comp. Therefore, the PDBj PubChem Portal uses RDKit to canonicalize both PubChem and PDB chem_comp entries for consistent matching, enabling accurate atom‐level alignment and interaction mapping. This alignment establishes a direct link between chemical compounds and their structural contexts in the PDB. The resulting connections are stored in our Mine RDB (via pdbj.link_entity_pdbjplus and cc.link_entry_pdbjplus) and are also available in downloadable mmJSON files.

**FIGURE 1:**
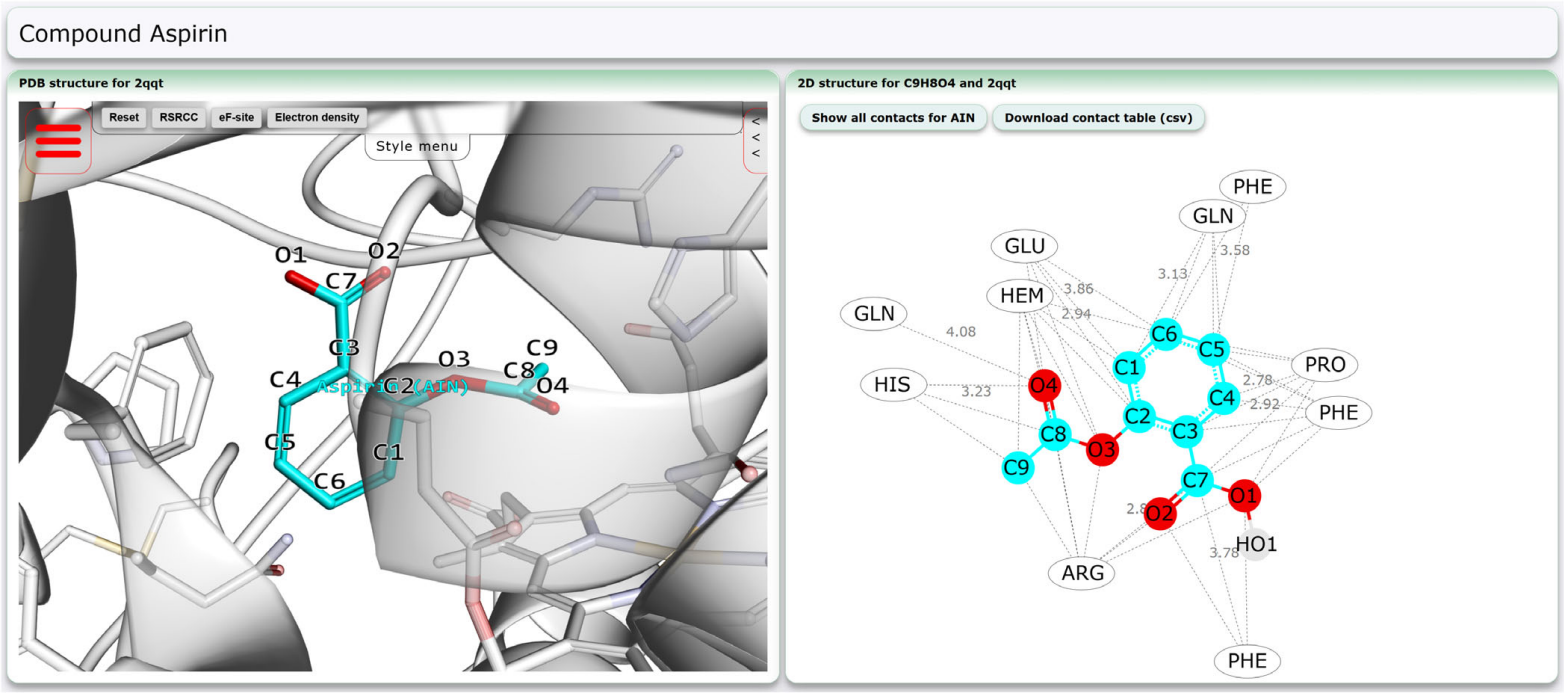
PubChem portal three‐dimensional (3D) and 2D structure panels. The top panel shows the name of the compound. The left panel displays a 3D molecular view of a template Protein Data Bank (PDB) entry (selected based on the highest Real‐Space Correlation Coefficient [RSRCC] value, or resolution if no entries with RSRCC values are available), with the ligand centered and atom labels shown. Nearby protein side chains are rendered as semi‐opaque stick models, and the protein is displayed as a semi‐opaque cartoon. Above the molecular view, a menu bar provides visualization options to color residues by RSRCC values, display electrostatic surfaces from our eF‐site service, overlay electron density contours from our EDMap service, or color residues based on dynamics from our Dynamics DB service for PDB entries with corresponding data. The right panel presents an interactive 2D representation of the ligand and its surrounding residues, with atom labels and clickable atoms that highlight the corresponding positions in the 3D structure. Also shown are the contacts each residue forms with the ligand as dotted lines, with the residue‐compound distance listed.

**FIGURE 2:**
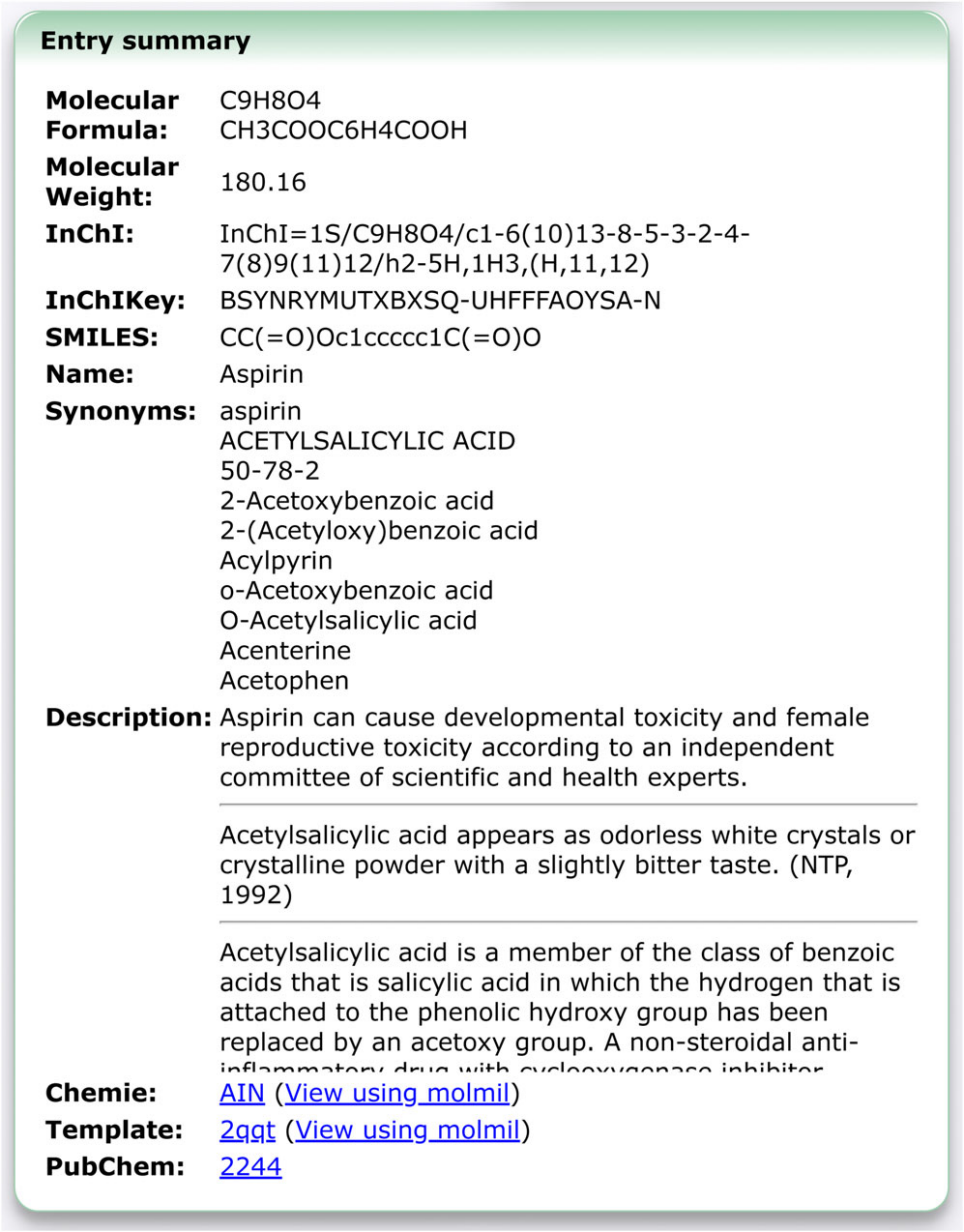
PubChem portal entry summary panel. This panel provides a comprehensive overview of the compound's chemical properties, including molecular formula, molecular weight, IUPAC International Chemical Identifier (InChI) and SMILES codes, name, synonyms, and a description. It also includes links to view the compound in Molmil and to the corresponding Protein Data Bank entry.

**FIGURE 3:**
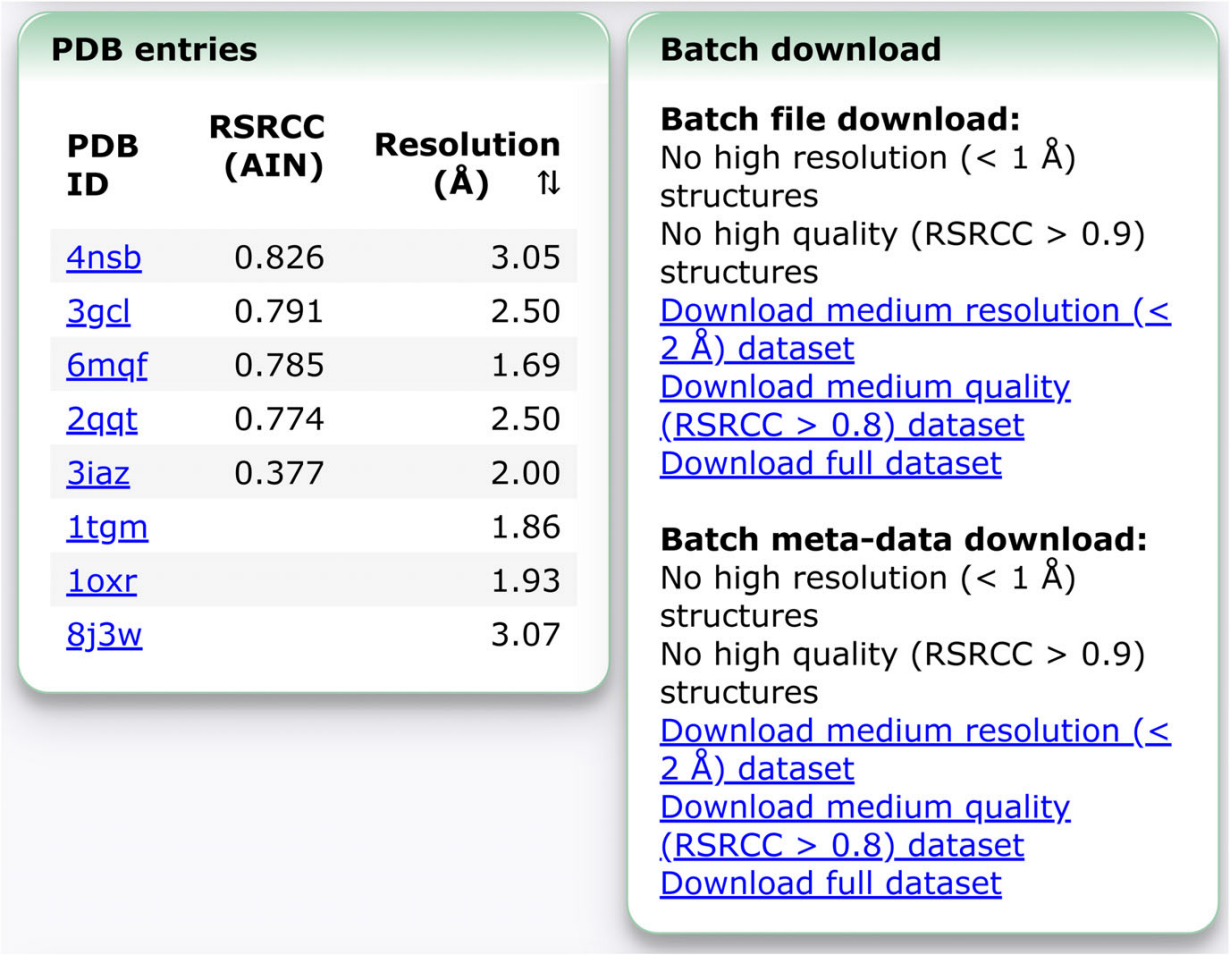
PubChem portal Protein Data Bank (PDB) entry and batch download panels. The left panel lists all PDB entries for the compound, filtered by protein class (UniProt or EC number) and sorted by Real‐Space Correlation Coefficient (RSRCC) value by default (with a secondary sort by resolution for entries lacking RSRCC values), or alternatively by resolution in Å. A batch download option in the right panel enables users to retrieve either the full or filtered dataset in various formats (e.g., mmCIF, mmJSON, legacy PDB) or as metadata from the Protein Data Bank Japan Mine database, categorized by RSRCC (high: >0.9, medium: >0.8), resolution (high: <1 Å, medium: <2 Å), or all entries.

**FIGURE 4:**
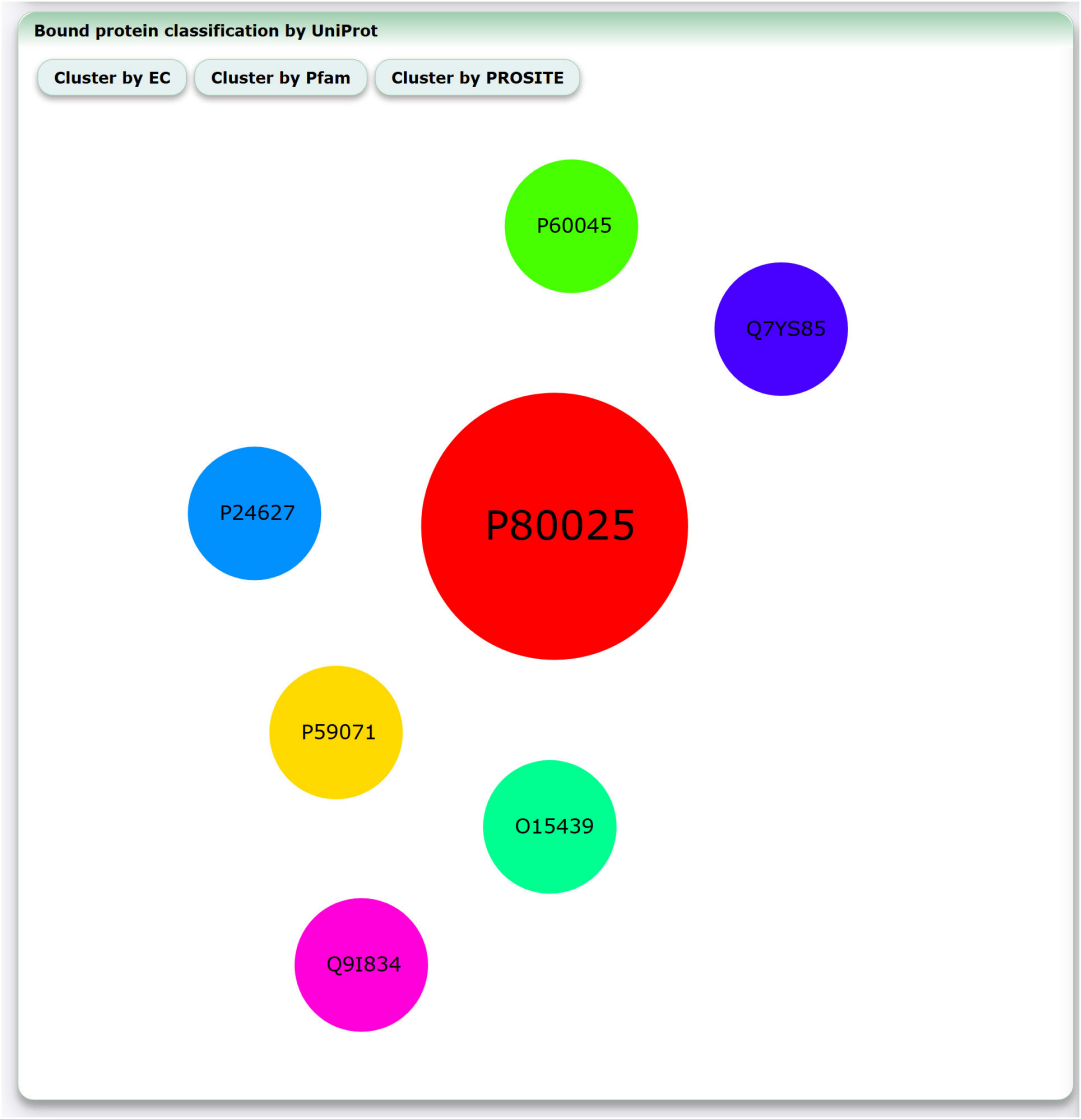
PubChem portal bound protein classification panel. This panel classifies the proteins bound to the compound by UniProt ID, EC number, Pfam ID, or PROSITE ID, with clusters sized proportionally to the number of entries. Clicking on a cluster filters both the Protein Data Bank entries and the template structure to display only those relevant to the selected protein class, enabling focused exploration of ligand–protein interactions.

The new service integrates both chemical data from PubChem and structural data from the PDB, with links to the PubChem portal pages accessible from two primary entry points. For PDBj Mine, users can find these links in the “Entities” panel (found within the Structural details tab). In this table's “DB Name” column, entities corresponding to chemical compounds display direct hyperlinks to both the Chemie database and the PubChem Portal page, if available. Similarly, for Chemie, the right‐hand side of any chem_comp entry page features an “External information” panel that lists links to matched databases, including a direct link to the PubChem Portal entry page, if available. Additionally, a top page that describes the service and enables searching by PubChem ID is available at https://pdbj.org/pubchem. Figures 1, 2, 3, 4 and S1 show parts of the portal page for aspirin (PubChem ID 2244). For each entry, a template PDB entry is shown in the left interface panel (Figure 1), visualized using our molecular viewer, Molmil. This template is selected as the PDB entry with the highest Real‐Space Correlation Coefficient (RSRCC), falling back to resolution if no structures with RSRCC values are available. Approximately 76% of PDB entries carry RSRCC data (as of May 2026); for the remaining cases, we use the global resolution metric as a fallback. The RSRCC measures the local fit between the ligand and its experimental electron density (values closer to 1 indicate a better fit). We prioritize this metric because it specifically identifies poorly‐fit ligands even in high‐resolution structures, which a global resolution metric might miss. Therefore, focusing on model quality surrounding the ligand is preferred over relying solely on overall structure resolution. The ligand within the PDB entry is centered in the screen, with atom names labeled; nearby side chains from interacting protein molecules are displayed as semi‐opaque stick models, and the protein as a semi‐opaque cartoon. The right panel shows an interactive 2D representation of the compound and the surrounding residues in the template structure (Figure 1). Here, the atom names as found in the PDB, which often differ from those in other databases, are also labeled; clicking on one of these atoms highlights the corresponding atom in the 3D structure panel on the left. At the top of the panel, several visualization options, using data from our secondary databases, are listed. If available, electrostatic surfaces from our eF‐site service, electron density contours from our EDMap service, and MD‐derived dynamics data from our Dynamics DB, which was recently updated to include protein–ligand complexes, can be displayed within the viewer. The integration of protein–ligand complex data into Dynamics DB enables comprehensive stability analysis of ligand‐bound conformations, offering valuable insights into binding dynamics and potential off‐target interactions.

We previously introduced a contacts table in our RDB that lists all intermolecular contacts between proteins and their partners (Bekker et al., 2022). We used this data in our earlier work to build a 2D topology viewer that indicates the intermolecular interactions between molecules in a PDB entry (Bekker et al., 2025a). Here, we have extended the contacts table to include intermolecular interactions between ligands and to indicate which atom pairs are in close proximity. Using this information, we have extended the ligand 2D representation to show nearby protein side chains and their approximate locations, providing an idea of the interactions formed between the ligand and its partner molecules. Here, interaction patterns, that is, the interactions formed between a side chain and the atoms of the ligand, are indicated by dotted lines, with residue‐compound distances displayed. Since a single compound may have many partner molecules, we also provide a feature to display all contacts for the given compound. This offers an overview of all interactions that the compound forms within the PDB, along with their approximate locations, enabling an assessment of potential binding modes. Knowledge of interaction patterns in the PDB may guide mutational studies to improve compound affinity or assist in crystallization by providing structural insights to enhance binding or identify compounds that could improve stability. For instance, by identifying rare but stable interactions across multiple entries, users can design chimeric structures to optimize ligand binding. While this analysis can be performed directly from the interface, the contacts table is also accessible via our REST services as part of the Mine RDB. This allows researchers to design custom logic, scan large numbers of complexes, or train machine learning models. In the interface, when displaying all interactions for a compound, interaction patterns from multiple PDB entries are clustered to identify recurring motifs. Interaction patterns are defined as residue‐specific interaction profiles (residue name and list of interacting ligand atoms) extracted from the intermolecular contact table in the Mine RDB. Patterns are clustered using exact‐matching of residue‐atom interaction profiles, meaning only patterns with identical interactions are merged. These clusters are weighted by frequency across the PDB, with common patterns rendered more opaque to highlight prevalent binding features, ensuring both frequent, biologically significant interactions and rare, novel ones are clearly visible. In the interface, moving the cursor over one of these clustered residues reveals the list of PDB entries (with chain and residue IDs), while clicking on them copies the data to the clipboard, where users can then copy this to a document to accumulate and analyze the interaction data in a user‐curated manner, or download all the data as a comma‐separated values (CSV) file.

Below the two structural panels is the Entry summary panel (Figure 2), which lists various information about the compound. Most of the information in this panel is derived from the PubChem entry. This includes the molecular formula, molecular weight, InChI code, SMILES code, name, synonyms, and a description of the compound. In addition, links to Chemie and the template PDB are provided, as well as links to view them with Molmil. On the next row, PDB entries for the chemical compound are listed (Figure 3), showing the filtered list if entries are filtered by UniProt ID, EC number, Pfam ID, or PROSITE ID (see the next paragraph), along with the ligand's RSRCC value and the structure resolution. To the right, the batch download panel is shown (Figure 3), which provides options to download the dataset (either the full or filtered one), either as compressed archives containing multiple files (e.g., mmCIF, mmJSON, legacy PDB, etc.) or specific metadata obtained directly from our Mine RDB (available in mmCIF, mmJSON, CSV or JSON files). Here, the datasets are split into five types: high quality (RSRCC >0.9), medium quality (RSRCC >0.8), high‐resolution (<1 Å), medium‐resolution (<2 Å), and all entries, to allow users to prepare datasets, while also filtering on either local quality or full structure resolution.

In the final row, there are two more panels. The right panel displays the bound protein classification (Figure 4). Since the contact table in the Mine RDB contains a list of all interactions as well as all metadata from the PDB (in addition to our own annotations and external database linkage), this enables extraction of all interactions and classification based on multiple parameters. In this panel, classification can be performed by UniProt ID, EC number, Pfam ID, or PROSITE ID. The clusters are sized according to the number of entries in each cluster, and clicking on them opens the PubChem portal page for that cluster, with PDB entries filtered according to the selected UniProt ID, EC number, Pfam ID, or PROSITE ID. The filtering also affects the selected template entry, where a representative PDB entry is chosen based on the entries in the filtered list. Clicking on one of the clusters reloads the interface with the filter applied. Here, a link to the corresponding filter's entry at PDBj's UniProt Portal, Expasy's enzyme database, EBI's Pfam or Expasy's PROSITE is provided under the compound name at the top of the page. After filtering, the 2D structure panel allows the user to view only the interactions relevant to the selected protein class, rather than all bound proteins in the PDB. This enables users to compare different interaction patterns observed across distinct protein classes, which could provide insight into viable mutations from a broader structural perspective. For instance, identifying whether specific mutations are feasible given pocket compatibility constraints that might preclude them in other contexts.

To the right of the classification panel is the Database Information panel (Figure S1), which lists links to various external databases, as provided in the PubChem entry, and here the compounds can be studied more in‐depth in more specialized databases. For PubChem entries without PDB data, the displayed information and panels differ from those with PDB data (Figure S2). For these entries, at the top, the Entry Summary panel is displayed; below it, the Database Information panel (left) and the 2D structure panel (right) are shown, where the latter is static and rendered by RDKit. While PDBj caches the PubChem JSON files for the chem_comp linked entries and adds additional data to them, for the PubChem entries without PDB data, the PubChem JSON files are fetched on‐the‐fly from PubChem and no additional data is added.

## 2 CONCLUSION

By integrating raw experimental data, computational models, and derived biological insights, PDBj is uniquely positioned among wwPDB partners to systematically collect and preserve structural biology data across all experimental modalities and computational sources. This comprehensive, long‐term approach ensures data integrity, enhances scientific reproducibility, and enables both foundational research and advanced structural analysis. Through its robust suite of tools, ranging from centralized databases like PDBj Mine to interactive viewers such as Molmil and specialized services like the UniProt and PubChem Portals, PDBj provides a cohesive, user‐friendly, and increasingly intelligent ecosystem for exploring protein and compound structures. These services not only support direct structural interrogation but also bridge sequence, genomic, and chemical data, with the PubChem Portal specifically enabling the novel exploration of compound‐protein interaction landscapes, enabling researchers to make informed decisions in structure selection, experimental design, and drug discovery. By continuously responding to user needs through iterative integration with external databases and advanced visualization tools, PDBj remains at the forefront of structural biology informatics, driving innovation and accelerating discovery across the scientific community.

## AUTHOR CONTRIBUTIONS

**Gert‐Jan Bekker:** Conceptualization; methodology; software; visualization; writing – original draft; writing – review and editing; resources; validation. **Satomi Niwa:** Writing – review and editing; validation. **Chioko Nagao:** Data curation; writing – review and editing; validation. **Genji Kurisu:** Conceptualization; funding acquisition; writing – review and editing; supervision; validation.

## CONFLICT OF INTEREST STATEMENT

The authors declare no potential conflict of interest.

## Supporting information


**Data S1.** Figure S1 shows the Database Information panel linking to external databases like ChemSpider, ChEMBL, and DrugBank for enhanced chemical and biological context, while Figure S2 demonstrates the portal's adaptability by displaying a static 2D structure and basic information for PubChem entries without PDB structural data.

## Data Availability

Data sharing not applicable to this article as no datasets were generated or analysed during the current study.
