# Supplementary material for: Protein Data Bank Japan: A unified portal for integrating structural and chemical data to explore protein–ligand interactions in PDB and PubChem
Source: Protein Sci. 2026 Jul 8;35(8):e70702. doi: 10.1002/pro.70702 (PMC13346351; doi:10.1002/pro.70702)
Supplement: Supplementary file 1 — Data S1. Figure S1 shows the Database Information panel linking to external databases like ChemSpider, ChEMBL, and DrugBank for enhanced chemical and biological context, while Figure S2 demonstrates the portal's adaptability by displaying a static 2D structure and basic information for PubChem entries without PDB structural data. [file PRO-35-e70702-s001.pdf]

Protein Data Bank Japan: A Unified Portal for Integrating Structural and Chemical Data to Explore Protein-Ligand Interactions in PDB and PubChem

Gert-Jan Bekker,[*1,2] Chioko Nagao,[1] Satomi Niwa,[1] Genji Kurisu [1,3*]

1. Institute for Protein Research, University of Osaka, 3-2, Yamadaoka, Suita, Osaka 565-0871, Japan.
2. JBiC Research Institute, Japan Biological Informatics consortium (JBiC), TIME24 Bldg. 10F, 2-4-32, Aomi, Koto-ku, Tokyo 135-8073, Japan.
3. Protein Research Foundation, Ina 4-1-2, Minoh, Osaka 562-8686, Japan.

CORRESPONDING AUTHORS:

Gert-Jan Bekker (Email: gertjan.bekker@protein.osaka-u.ac.jp)

Genji Kurisu (Email: gkurisu@protein.osaka-u.ac.jp)

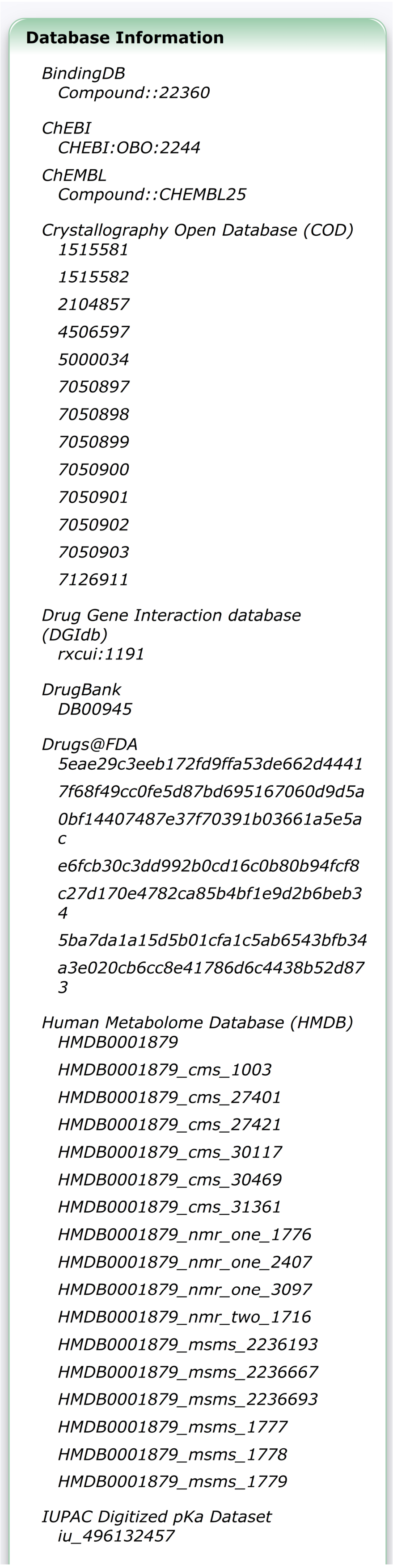

Figure S1. PubChem portal Database information panel. This panel provides links to external databases referenced in the PubChem entry, such as ChEMBL, DrugBank, COD, CCDC, and KEGG; the specific databases displayed vary depending on the compound. This enables users to access additional biological and chemical context for the compound.

Compound Adenosine 5'-(trihydrogen diphosphate), P'->5'-ester with 3-(aminocarbonyl)-1-beta-D-ribofuranosylpyridinium, inner salt, ion(1-)

Entry summary

Molecular Formula: C21H26N7O14P2-

Molecular Weight: 662.4

InChI: InChI=1S/C21H27N7O14P2/c22-17-12-19(25-7-24-17)28(8-26-12)21-16(32)14(30)11(41-21)6-39-44(36,37)42-43(34,35)38-5-10-13(29)15(31)20(40-10)27-3-1-2-9(4-27)18(23)33/h1-4,7-8,10-11,13-16,20-21,29-32H,5-6H2,(H5-,22,23,24,25,33,34,35,36,37)/p-1/t10-,11-,13-,14-,15-,16-,20-,21-/m1/s1

InChIKey: BAWFJGJZGIEFAR-NNYOXOHSSA-M

SMILES: NC(=O)c1ccc[n+]([C@@H]2O[C@H](COP(=O)([O-])OP(=O)([O-])OC[C@H]3O[C@@H](n4cnc5c(N)ncnc54)[C@H](O)[C@@H]3O)[C@@H](O)[C@H]2O)c1

Name: Adenosine 5'-(trihydrogen diphosphate), P'->5'-ester with 3-(aminocarbonyl)-1-beta-D-ribofuranosylpyridinium, inner salt, ion(1-)

Synonyms: DTXSID201016357
76961-04-1
RefChem:1076176
DTXCID301474532
Adenosine 5'-(trihydrogen diphosphate), P'->5'-ester with 3-(aminocarbonyl)-1-beta-D-ribofuranosylpyridinium, inner salt, ion(1-)
NAD+
DPN-ox
DPN+
nchembio867-comp14
NAD anion

Description: NAD(1-) is an anionic form of nicotinamide adenine dinucleotide arising from deprotonation of the two OH groups of the diphosphate moiety. It has a role as a cofactor, a Saccharomyces cerevisiae metabolite and a human metabolite. It is a hydrogen acceptor and an organophosphate oxoanion. It is a conjugate base of a NAD(+).

PubChem: 15938971

Database Information

ChEBI
CHEBI:OBO:15938971

2D structure for C21H26N7O14P2-

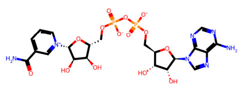

Figure S2. PubChem portal information displayed for entries without PDB data. For compounds lacking structural data in the PDB, the interface shows only the Entry Summary and Database Information panels (on the left) and a static 2D structure rendered by RDKit (on the right), highlighting the portal's adaptability to both PubChem entries with experimental structural data and those without.
